# Adjunctive daily supplementation with encapsulated fruit, vegetable and berry juice powder concentrates and clinical periodontal outcomes: a double-blind RCT

**DOI:** 10.1111/j.1600-051X.2011.01793.x

**Published:** 2012-01

**Authors:** Iain L C Chapple, Michael R Milward, Nicola Ling-Mountford, Paul Weston, Kevin Carter, Keeley Askey, Gerard E Dallal, Silke De Spirt, Helmut Sies, Dina Patel, John B Matthews

**Affiliations:** 1 Periodontal Research Group, School of Dentistry, College of Medical and Dental Sciences, University of BirminghamBirmingham, UK; 2 Gerald J & Dorothy R Friedman School of Nutrition Science and Policy, Tufts UniversityBoston, MA, USA; 3 Institute of Biochemistry and Molecular Biology I, Faculty of Medicine, Heinrich-Heine-UniversityDusseldorf, Germany; 4 College of Science, King Saud UniversityRiyadh, Saudi Arabia; 5 UK National External Quality Assessment (NEQAS) for Immunology, Immunochemistry and Allergy, Department of Immunology, Northern General HospitalHerries Road, Sheffield, UK

**Keywords:** berry, dietary intervention, fruit, Juice Plus+, micronutrient, periodontitis, randomized controlled trials, therapy, vegetable

## Abstract

**Aim:**

A double-blind randomized controlled trial to determine whether dietary supplementation with fruit/vegetable/berry juice powder concentrates, simultaneously with non-surgical periodontal therapy, improved 2-month treatment outcomes.

**Methods:**

Volunteers with chronic periodontitis were randomly assigned to one of three groups: fruit/vegetable (FV), fruit/vegetable/berry (FVB) or placebo. Supplements were taken daily during non-surgical debridement and maintenance and outcomes assessed at 2, 5 and 8 months after completion. Primary outcomes were mean probing pocket depth (PPD), clinical attachment gain, % sites bleeding on probing (% BOP) at 2 months. Adherence and plasma β-carotene were determined.

**Results:**

Sixty-one nutritionally replete (by serum biochemistry) volunteers enrolled and 60 (*n* = 20 per arm) completed the 2-month review. Clinical outcomes improved in all groups at 2 months, with additional improvement in PPD *versus* placebo for FV (*p* < 0.03). Gingival crevicular fluid volumes diminished more in supplement groups than placebo (FVB; *p* < 0.05) at 2 months, but not at later times. The % BOP (5 months) and cumulative plaque scores (8 months) were lowered more in the FV group (*p* < 0.05).

**Conclusions:**

Adjunctive juice powder concentrates appear to improve initial pocket depth reductions in nutritionally replete patients, where plasma micronutrient bioavailability is attainable. Definitive multicentre studies in untreated and treated patients are required to ascertain the clinical significance of such changes.

Periodontitis is a complex chronic inflammatory disease requiring the emergence of a pathogenic biofilm whose expression is governed by a number of host-dependent component causes. The qualitative and quantitative contributions of the various component causes to disease initiation and/or progression varies from patient to patient, resulting in a widely heterogenic clinical phenotype. Disease severity and extent also appear to be influenced by the same exposures, in conjunction with local anatomical features. Tissue destruction arises largely from a dysregulation of chronic inflammatory processes that the body's homoeostatic maintenance systems fail to resolve, resulting in a failure to eliminate pathogenic components of the subgingival biofilm and a persistence of chronic non-resolving inflammation (Van Dyke 2008).

Exposure categories believed to contribute component causes to periodontal inflammation include: genetic (Michalowicz et al. 1991); environmental (e.g. biofilm, stress); lifestyle/behavioural (e.g. smoking, diet) and pharmacological (e.g. corticosteroids); and “nutrition”, which is influenced by lifestyle, environmental and genetic exposure categories (Chapple 2009). Evidence is emerging for associations between such lifestyle factors and periodontal inflammation (Bawadi et al. 2011).

Epidemiological studies demonstrate that a higher intake of fruit and vegetables (FV) is associated with a lowered risk of atherogenic cardiovascular disease (Joshipura et al. 1999), ischaemic stroke (Joshipura et al. 2001) and mortality (Knekt et al. 1996, Sauvaget et al. 2003). Diets rich in vegetables and Vitamin C also appear to associate positively with better periodontal health and negatively with periodontal disease progressing more rapidly in undernourished populations (Enwonwu et al. 2002). Given the high prevalence of periodontitis in the population (moderate disease prevalence 20–50%; König et al. 2010) and its impact upon elevating plasma biomarkers of systemic inflammation (D'Aiuto et al. 2004a, b), it appears that periodontal inflammation makes a significant contribution to the systemic inflammatory burden.

In recent years, strong evidence has emerged that diets rich in refined carbohydrates and saturated fats are pro-inflammatory, whereas those rich in polyunsaturated fats (fish oils), antioxidant micronutrients (fruits, berries and vegetables) and certain nuts (cashews) are anti-inflammatory (O'Keefe et al. 2008). Pro-inflammatory diets drive oxidative stress within cells and tissues through metabolic (mitochondrial) and receptor-mediated pathways (reviewed by Chapple 2009) and such post-prandial oxidative stress (Sies et al. 2005), termed “meal induced inflammation” (O'Keefe & Bell 2007) positively correlates with the magnitude (Esposito et al. 2008) and frequency (Ceriello et al. 2008) of post-meal surges in glucose and triglycerides. The latter, a consequence of the rapid absorption of glucose and lipids into the blood stream following intake of dietary refined carbohydrate and saturated fats, gives rise to elevations in plasma levels of CRP and pro-inflammatory cytokines (Monnier et al. 2006) and oxidative stress (Chapple 2009). Indeed, recent evidence has demonstrated a significant role for oxidative stress in promoting bone resorption via activation of certain transcription factors (FoxOs, which decreases wnt signalling), modulated by insulin resistance and increasing age (reviewed by Galli et al. 2011). Antioxidant micronutrients combat such pro-inflammatory cascades through modulation of oxidative stress by directly scavenging reactive oxygen species (ROS) and also by down-regulation of some redox-sensitive pro-inflammatory gene transcription factors such as nuclear factor-kappa B and activator protein-1 while up regulating anti-inflammatory gene transcription factors such as nuclear factor (erythroid-derived 2)-like 2 (Nrf2: reviewed by Chapple & Matthews 2007). Indeed, adjunctive phytonutrient supplementation using juice powder concentrates of fruit, vegetables and berries (FVB) has been shown to reduce the immediate impact of a high fat test meal upon inflammatory biomarkers and to improve flow mediated dilatation of the brachial artery (Plotnick et al. 2003).

Oxidative stress, defined as *an imbalance between oxidants and antioxidants in favour of the oxidants, leading to a disruption of redox signalling and control and/or molecular damage* (Sies & Jones 2007) is a key pathological event underpinning periodontal tissue destruction (Chapple & Matthews 2007, Bullon et al. 2009, Galli et al. 2011). Since the first indirect demonstration of oxidative stress within the periodontal tissues (Chapple et al. 1997), a body of evidence has accumulated demonstrating elevated biomarkers of oxidative stress (Sugano et al. 2000, Takane et al. 2002, Wei et al. 2004, Panjamurthy et al. 2005, D'Aiuto et al. 2010) and antioxidant compromise (Chapple et al. 1997, 2002, Brock et al. 2004, Panjamurthy et al. 2005, D'Aiuto et al. 2010) in periodontitis patients. Recent data from our own laboratory demonstrated that the antioxidant depletion manifest locally within the periodontal tissues appears to be an effect rather than a cause of the oxidative stress, which develops during periodontal inflammation (Chapple et al. 2007, Grant et al. 2010). Nevertheless, it is biologically plausible that boosting the antioxidant micronutritional status of patients may have preventive and/or adjunctive therapeutic benefit, in particular in those patients who show micronutrient deficiency (Tonetti & Chapple 2011).

The potential impact of diet upon periodontal inflammatory status was recently illustrated in a Swiss study, which found that when 10 adults were placed in a “stone-age” environment for 4 weeks, with negligible oral hygiene and diets were “stone-age” in nature (id est. low in simple sugars and high in antioxidant micronutrients, fish oils and fibre), they remarkably demonstrated significant decreases in gingival bleeding and probing depths, despite significant increases in plaque accumulation (Baumgartner et al. 2009).

Recent case–control studies have demonstrated that periodontitis patients have significantly lower serum antioxidant micronutrient levels than unaffected controls (Brock et al. 2004, Panjamurthy et al. 2005, Konopka et al. 2007), and also compromised gingival crevicular fluid (GCF) antioxidant status (Chapple et al. 2002, 2007b, Brock et al. 2004). Non-surgical periodontal therapy has been shown to restore certain antioxidant components in a process attributed to reducing levels of oxidative stress secondary to the resolution of periodontal inflammation (Chapple et al. 2007). However, non-surgical therapy did not restore levels of the important small molecule antioxidant species, glutathione (GSH), although the ratio of GSH to its oxidized counterpart was restored, implying at least a restoration of the redox balance within periodontal cells and tissues following successful periodontal therapy (Grant et al. 2010). Large scale epidemiological studies also consistently support a strong inverse association between serum antioxidant micronutrient concentrations and periodontitis prevalence and severity, in several different populations (Nishada et al. 2000, Amarasena et al. 2005, Amaliya et al. 2007, Chapple et al. 2007, Linden et al. 2009). Jenzsch et al. (2009) demonstrated that diets rich in vegetables, fruits, legumes and dairy products, when employed as the only intervention, significantly improved pocket depth and gingival inflammation in periodontitis patients with metabolic syndrome. The reported data point towards the biological plausibility of beneficial periodontal outcomes being derived from phytonutritional interventions of antioxidant micronutrients, acting by direct scavenging of ROS, and also by modulation of redox-sensitive pro-inflammatory gene transcription factors.

There remains a paucity of data from prospective intervention studies on the role of nutrition in the pathogenesis of periodontitis (van der Velden et al. 2011). Specifically, there are no reports from placebo-controlled randomized trials on the efficacy of nutritional supplements upon periodontal outcomes. Therefore, we hypothesized that:

- Daily supplementation with a primarily antioxidant juice powder phytonutrient (Juice Plus+®-FV) would significantly improve treatment outcomes over placebo supplementation, at 3 months post-therapy commencement (2 months post-therapy completion), when used as an adjunct to conventional non-surgical periodontal therapy.
- Triple therapy with the above supplement (Juice Plus+®-FVB) would produce additional treatment benefit over dual therapy (FV) and over placebo.
- Improved treatment outcomes with the supplement will be maintained at 9 months post-therapy commencement.

The aim of this preliminary investigator-led study was to ascertain whether or not daily dietary supplementation with capsules containing primarily FVB juice powder concentrates (Juice Plus+®; NSA LLC, Collierville, TN, USA), taken during standard non-surgical periodontal therapy (no pre-dosing), improved clinical outcomes of periodontal treatment 2 months following completion of non-surgical therapy (simultaneous supplementation).

## Material and methods

### Study ethics and governance

This was an investigator-led, three-arm, placebo-controlled, double-blind randomized intervention study (NCT00952536) and was approved by South Birmingham Local Research Ethics Committee (05/Q2707/252), and research governance was overseen by South Birmingham Primary Care Trust Research Management and Governance (Project Number – SouthDent116/742). The study was conducted in accordance with international Good Clinical Practice standards.

### Study design and volunteers

Volunteers (*n* = 61) were recruited from new patient consultation clinics by one study team member (P. W.), and were both non-smokers and medically healthy by medical history questionnaire. A rolling recruitment protocol was adopted and non-smokers selected, as smoking impacts upon oxidative stress (Chapple & Matthews 2007) and was deemed to be a likely confounder of study outcomes. Volunteers were aged between 30 and 60 years, either men or women, and had chronic periodontitis as defined by a minimum of two sites per quadrant with pocketing or interproximal attachment loss of >6 mm and one-third radiographic bone loss. The following exclusion criteria were applied: patients with aggressive disease, patients with physical or mental disability, pregnant women, patients whose medical history may place them at risk of complications from periodontal therapy (e.g. need for antibiotic prophylaxis, Warfarinised patients), patients taking long-term antimicrobial or anti-inflammatory drugs, patients unable to swallow capsules, patients unable to provide informed consent, current smokers (or within 5 years), patients taking regular vitamin supplementation. The study was designed to exit 60 volunteers at 9 months following recruitment (8 months following completion of non-surgical treatment) and was conducted over a 4.5-year period (2005–2010).

Volunteers were provided with a detailed information sheet and allowed 2 weeks to consider their participation, prior to obtaining their informed consent and enrolment. As volunteers were enrolled (by M. M.) they were allocated sequential study numbers (by K. A.) and provided with their capsules, sufficient for 3 months.

Randomization was performed by the study statistician (G. D.), who was independent of the clinical study team and site, using a computer-generated program http://www.randomization.com (volunteer numbers generated from 001 to 070 to allow for loss of up to 10 participants). The capsules were supplied in pre-labelled containers.

**Fig. 1:**
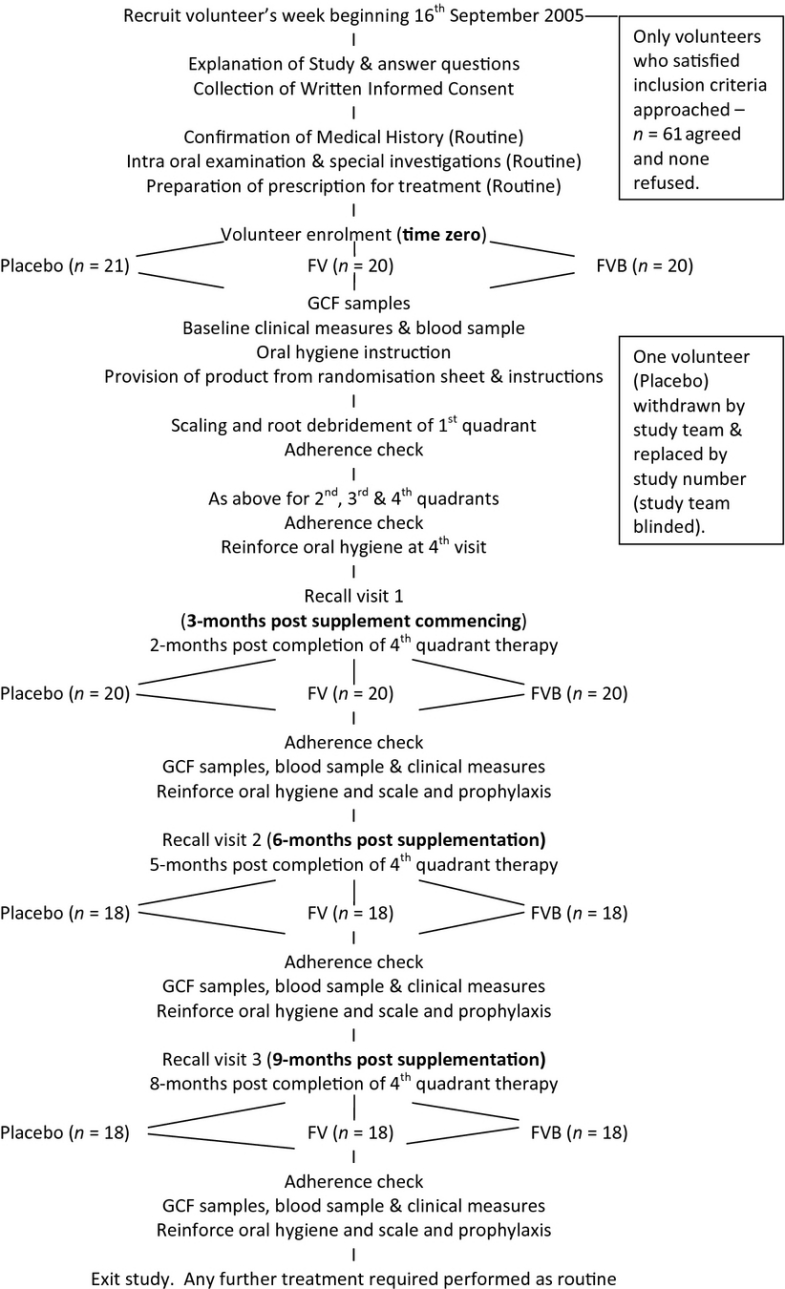
Study flow chart.

Baseline measures were also collected at the enrolment visit, prior to which volunteers were asked to fast overnight, not to brush and not to chew gum or drink within 2 h of their appointment. Protocol adherence was checked prior to sample collection, and patients were re-appointed if necessary. Biological samples were sequentially collected. The GCF from three deep sites (≥6 mm) and three shallow sites (≤3 mm) was collected as previously reported (Chapple et al. 2007) and deep sites were pooled, on a volunteer and visit basis, as were samples from the three shallow sites. The GCF sampling was followed by a venous blood sample, probing pocket depth (PPD) and recession measures, bleeding scores, gingival colour index [Modified Gingival Index (MGI) – Lobene et al. 1986] and plaque indices (Fig. 1). Patients then had scaling and root surface debridement performed on a quadrant by quadrant basis within 1 month and over four visits. At each visit, adherence was re-checked and recorded. Capsule re-supply occurred at recall visits and patients were recalled at 2, 5 and 8 months following completion of the last session of instrumentation. At each recall visit, clinical samples and measurements were repeated and volunteers exited the study at the 8-month recall appointment, when capsule containers were returned and counted. Volunteers were also asked to maintain a supplement diary recording of how many capsules had been missed. If further therapy was required at this stage, as judged by clinical examination, it was performed by the same therapist (N. L.-M.), but outside of the study.

All volunteers completed standard non-surgical periodontal therapy, which was performed by a single operator (N. L.-M.; 15 years experience and MPhil degree in clinical periodontal trials) within 4 weeks of commencing treatment and all indices were recorded by a single GCP-trained examiner (M. M.). Scaling and debridement was performed using a traditional quadrant by quadrant protocol, using a single ultrasonic (Densply CavitronPlus SPS; Densply UK) device and FSI-100 inserts, and Gracey curettes (LM-Dental UK). Treatment was performed systematically until the root surfaces were considered sufficiently clean, each quadrant taking 30–45 min.

Patients commenced their assigned capsule supplement at their study enrolment study visit, within 1 week of physical instrumentation commencing (Fig. 1) and continued supplementation during treatment (1 month) and also for 8 months of maintenance (total 9 months of supplementation). Compliance was checked by capsule count and also by analysing serum β-carotene levels. There were three study arms:

Test 1 – Six capsules daily containing the total equivalent of four FV capsules, and two placebo capsules during periodontal therapy and for 8 months thereafter.
Test 2 – Six FVB capsules daily during periodontal therapy and for 8 months thereafter.
Control *–* Six placebo capsules daily during periodontal therapy and for 8 months thereafter.

In all three study groups, capsules were taken with food twice daily (three in the morning, three in the evening).

### Outcome measures

Primary outcome measures were (1) reductions in PPDs (mean per patient reduction in PPD), (2) reduction in percentage sites bleeding on probing (% BOP) from the marginal tissues (as a primary outcome measure of tissue inflammation), and (3) mean increase in clinical attachment level (CAL) at the 2-month post-therapy recall visit (3 months post-therapy commencement). Secondary outcome measures were reductions in GCF volume from both deep and shallow sites, gingival redness (MGI – Lobene et al. 1986), cumulative plaque index (Lobene et al. 1982) and recession. Although not part of the original protocol, a decision was made to additionally express (4) pocket depth reductions as “number of residual sites >4 mm”, consistent with contemporary studies published subsequent to protocol preparation (Wennström et al. 2005).

Probing measures were performed in duplicate at six sites per tooth (two marginal and four proximal) with a constant force (UNC CP-15 markings – 0.2 N force) probe and where differences between duplicate measures of greater than 1 mm arose, a third measure was taken. The mean of the two closest measures was used. Mean probing attachment (derived from pocket depth + recession), and PPD measures per subject were calculated at each examination point (baseline, 2, 5 and 8 months post-therapy). Marginal bleeding was recorded dichotomously (four sites per tooth, mesial, distal, mid-facial, mid-lingual) and expressed as mean percentage sites bleeding on probing (% BOP) per subject at each examination point. Plaque levels were quantified using a modification of the Quigley-Hein index (Lobene et al. 1982) and expressed as a whole mouth total (cumulative) score per volunteer at each examination point. The study visits and procedures are summarized in Fig. 1. Outcomes at the 5- and 8-month recall visits were considered secondary outcomes, as pathogenic biofilm changes are evident at this stage (Quirynen et al. 2005) and may lead to early signs of recalcitrant disease.

### Biological samples collected

Gingival crevicular fluid samples were collected over 30 s using Periopaper™ (Oraflow Inc., Smithtown, NY, USA) strips from the mesio-buccal aspects of the three deep and three shallow molar sites per subject as previously described (Brock et al. 2004). Volumes were read on a pre-calibrated Periotron 8000™ according to standard methodologies (Chapple et al. 1999).

Blood was collected into Vacutainer™ (NHS supplies, Alfreton, Derbyshire, UK) tubes for serum and plasma preparation (*vide infra*). Platelet depleted plasma was prepared by centrifugation at 1000 *g* for 30 min. (4°C). Serum was aliquoted into cryogenic vials, snap frozen and stored at −80°C for subsequent analysis of β-carotene and vitamin E. For vitamin C assay, 0.75 ml metaphosphoric acid (100 g/l) was added to 0.75 ml plasma to precipitate proteins, prior to storage at −80°C. All samples were stored in the dark and kept free from direct sunlight at all stages of handling.

### Test products

The verum test capsules are marketed commercially as Juice Plus+® and contain a fine, granular powder, encapsulated in a size 00 gelatin capsule. The placebo test capsules were of identical appearance and contained primarily microcrystalline cellulose.

The FV capsule contents consisted primarily of a blended FV pulp and juice powder concentrate derived from Acerola cherry, apple, beet, beetroot, broccoli, cabbage, carrot, cranberry, dates, garlic, kale, orange, peach, papaya, parsley, pineapple, prune, spinach, sugar beet, tomato, with *Spirulina pacifica*, *Lactobacillus acidophilus*, rice bran, oat bran and *Dunaliella salina*. These active ingredients were supplemented to provide declared totals (per daily dose) of β-carotene (7.5 mg), vitamin E (46 mg), vitamin C (200 mg) and folic acid (400 μg). Although the phytonutrient capsules are known to contain polyphenolic antioxidant micronutrients, these vary according to growing and harvest conditions and absolute levels were not analysed.

The FVB capsule contents consisted primarily of a blended fruit, berry and vegetable pulp and juice powder concentrate. In addition to all the components within the FV capsules, FVB capsules also contained material derived from blackberry, black currant, blueberry, bilberry, concord grape, elderberry, raspberry, red currant, green tea, ginger root, artichoke leaf, grape seed extract in addition to arginine, carnitine and co-Enzyme Q10. These active ingredients were supplemented to provide declared totals (per daily dose) of β-carotene (7.5 mg), vitamin E (66 mg), vitamin C (222 mg) and folic acid (640 μg). Small quantities of anti-caking agents (calcium carbonate, magnesium oxide, magnesium stearate, silicon dioxide, cellulose) and thickeners (citrus pectin, guar gum) are added to assist the manufacture of capsules.

### β-Carotene analysis (for adherence)

Adherence to supplement usage was assessed by residual capsule counts and a supplement adherence diary at recall visits and also biochemically by analysis of serum β-carotene levels. β-carotene was determined by HPLC using isocratic elution, a reversed phase column (pKb-100, 250 × 4.6 mm^2^; Supelco, Bellefonte, PA, USA), protected by a guard column (4.6 × 4.6 mm²) with the same stationary phase and a Merck-Hitachi L-7100 pump connected with a Merck-Hitachi UV/Vis detector (Merck-Hitachi, Darmstadt, Germany). β-carotene was detected at 450 nm and the concentration calculated from external calibration curves generated with original standard compounds and internal standards as previously reported (Stahl et al. 1993).

### Plasma vitamin E and vitamin C determination at baseline

These analyses at baseline were performed to assess whether or not any of the volunteers were vitamin deficient. Alpha-tocopherol was extracted and detected simultaneously with the UV/VIS detector set at 292 nm (Polidori et al. 2001). Vitamin C was determined by HPLC using a Merck- Hitachi L-6200, a pump connected with a Merck- Hitachi UV/Vis detector (Merck-Hitachi) and using a commercially available analytical kit (Chromsystems Instruments & Chemicals GmbH, Munchen, Germany), run according to manufacturer's instructions.

### Power calculation and statistical analysis

The reported study was the first of its type to the authors’ knowledge and therefore a power calculation based upon mean (±SD) outcomes from pilot work was not possible for antioxidant outcomes. However, using the primary outcome measure of reduction in PPD and assuming a mean additional PPD reduction in the supplement groups of 0.4 mm (as this has been demonstrated in pharmacological interventions) over non-surgical therapy alone, 17 volunteers per treatment group were needed to complete the study for a two-sided test of equality of means at the 0.05 level of significance with 80% power. This was based on accepted mean PPD reductions for mild to moderate periodontitis of 1 mm (moderate) to 2 mm (deep sites) with non-surgical therapy, and also upon data from a previous study that demonstrated mean PPD reductions of 1.1 ± 0.4 mm (Chapple et al. 2007). The study was powered to detect differences between test and placebo groups only. A total of 61 volunteers were sequentially recruited.

Statistical analysis was performed by the study statistician (G. D.) and utilized analysis of covariance, with baseline measures employed as the covariates. Tukey's Honestly Significant Difference was used to compare the three groups. Analyses were performed using SAS for Windows, version 9.2 (SAS Institute Inc., Cary, NC, USA). All tests were two-sided and a result was judged statistically significant if its observed significance level (*p*-value) was less than 0.05. An intention-to-treat (ITT) analysis was performed, employing data from all subjects regardless of adherence to treatment. Serum β-carotene concentrations were also used as a biochemical measure of adherence and/or an indirect measure of carotenoid bioavailability.

Missing data: The primary outcome measures were taken as part of standard care during the subjects’ 8-month review. There was no reason for missing data to be related to treatment or outcome, but missing data were checked for any relationship to treatment assignment. Otherwise missing values were ignored in the analyses because they were deemed to be missing completely at random.

Code breaking: The code was retained by the statistician and not broken until the last patient had completed their 8-month recall visit, and all data analysis had been completed.

## Results

### Volunteer flow through study

The flow of patients through the study stages is illustrated in Fig. 1. Sixty-one patients were enrolled and sixty completed the 2-month post-therapy visit, providing primary outcomes for 20 volunteers in each study group (FV, FVB and placebo). Six patients withdrew at the 2-month post-therapy visit. The reasons for withdrawal were: capsules too large to swallow, moved away from area, difficulty attending appointments, unrelated medical problems, lost to follow-up for unknown reason. One patient was withdrawn (placebo group) as they telephoned to inform that they were experiencing difficulty swallowing, and although this could not be attributed to the supplements as opposed to a respiratory tract infection, the principal investigator (I. L. C.) decided to withdraw the volunteer by telephone and to complete treatment outside of the study. This last volunteer was replaced with the 61st enrolee. To ensure groups remained balanced, whilst at the same time maintaining the blinding of the study, the statistician provided a replacement study number.

### Demographical and baseline nutritional data

The volunteer demographics are recorded in Table 1, and although more women were enrolled than men, the groups were similar with regard to age and gender. There were no baseline differences between groups in peripheral blood levels of vitamin C (range = 48–58 μmol/l) and vitamin E (range = 17–19 μmol/l), which were within SI reference ranges (Table 1). Baseline β-carotene concentrations (range = 0.5–0.7 μmol/l) were below the low end of one reference range (Young 1987), but within another commonly used range (Iverson et al. 2007); however, there were no differences between the study groups.

**Table:** Demographical and baseline micronutrient data [mean (μmol/l) ± SD] of the test groups

Test group	No.	Men	Women	Age (years)		β-carotene* (0.9–4.6)† (0.2–1.6)‡	Vitamin C§ (30–110)† (23–85)‡	Vitamin E* (18–29)† (12–42)‡
							
				Mean ± SD	Range			
Fruit and vegetable	20	6 (30%)	14 (70%)	48.3 ± 8.4	35–69	0.7 ± 0.45	48.1 ± 16.2	18.3 ± 4.4
Fruit, vegetable and berry	20	8 (40%)	12 (60%)	48.1 ± 7.4	33–58	0.6 ± 0.3	57.4 ± 17.6	18.6 ± 5.9
Placebo	20	7 (35%)	13 (65%)	47.9 ± 6.6	38–60	0.5 ± 0.25	57.9 ± 22.0	17.2 ± 3.2

* serum.

§ plasma.

† SI reference ranges (μmol/l) (Young 1987).

‡ SI ranges from Iversen et al. AMA Manual of Style: a guide for authors and editors. 10th Edition, New York, Oxford University Press, 2007.

### Adherence/bioavailability of β-carotene

**Fig. 2:**
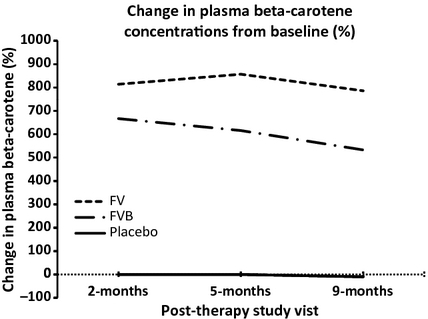
Percentage change in plasma beta-carotene concentrations from baseline.

The supplement diaries and capsule counts indicated that protocol adherence had been equivalent in all groups. Serum β-carotene concentrations increased significantly at 2 months in both FV and FVB groups (FV by 5.4 μmol/l, *p* < 0.0001; FVB by 3.5 μmol/l, *p* < 0.0001), but there was no change in the placebo group. The differences between the supplement and placebo groups post-supplementation were significant (*p* < 0.001) and the increase in serum β-carotene concentrations was highest in the FV group (Fig. 2) indicating greater micronutrient bioavailability in this group compared with the FVB group, which does not appear to result from between group differences in adherence, but might have impacted upon clinical outcomes.

### Changes in primary outcomes

**Fig. 3:**
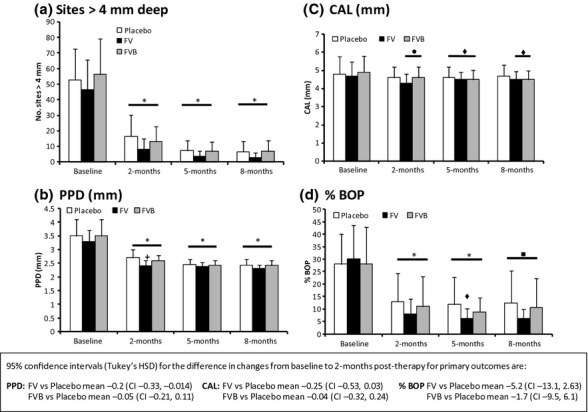
Changes in primary outcomes during the study period (mean ± SD). (a) Sites >4 mm deep; (b) PPD (mm); (c) CAL (mm); (d) %BOP. Bars represent comparisons with baseline. Symbols alone represent comparisons with placebo. *p < 0.0001; ▪*p* = 0.002; •*p* < 0.01; +*p* < 0.03; ♦*p* < 0.05.

The improvement in clinical outcomes in the placebo group was consistent with the literature (Cobb 2002) and statistically significant. This is evident from the statistically significant reduction of sites >4 mm at 2, 5 and 8 months post-therapy completion (Fig. 3a; *p* < 0.0001). Thus, after a single phase of non-surgical therapy, “closed pockets” (Wennström et al. 2005) increased in number from an average of 70% of sites in all three groups at baseline to 98% (FV group) and 91% (FVB and placebo groups).

Other primary outcome measures, pre- and post-completion of non-surgical scaling and root surface therapy, are illustrated in Fig. 3b–d (and mean values documented in Table S1). At 2, 5 and 8 months post-treatment completion there were significant reductions in mean PPD compared with baseline, consistent with literature reports for moderate periodontitis (Cobb CM 2002; van der Weijden & Timmermann 2002) for all three groups (*p* < 0.0001). At 2 months, reductions in PPD were statistically significantly greater in the FV supplement group relative to the placebo group (*p* < 0.03; Fig. 3b). This was not the case for the FVB group (*p* = 0.7; Fig. 3b). Although the PPD levels in the FV group remained lower than the placebo and FVB groups at 5- and 8-month recalls, differences were no longer statistically significant at these secondary time points.

Significant post-treatment CAL gains were detected at all time points in both FV (*p* < 0.05) and FVB (*p* < 0.02) supplement groups (Fig. 3c). In contrast, CAL gains in the placebo groups were only significant over baseline at 5 months. Despite this, CAL gains within supplement groups over those measured in the placebo group did not reach statistical significance (*p* > 0.09).

At all post-treatment reviews, the percentage of sites bleeding on probing (% BOP; Fig. 3d) was significantly reduced in all three study groups (*p* < 0.002). Although the reduction in % BOP was greatest at all time points for the supplement groups, the additional improvement relative to placebo was only statistically significant at the 5-month secondary time point for the FV group (*p* < 0.05).

### Changes in secondary outcomes

**Fig. 4:**
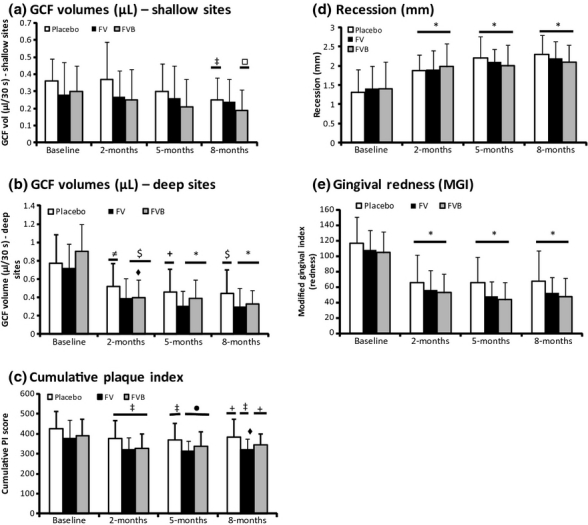
Changes in secondary outcomes during the study period (mean ± SD). (a) Gingival crevicular fluid (GCF) volumes (μl) – shallow sites; (b) GCF volume (μl) – deep sites; (c) cumulative plaque index; (d) recession (mm); (e) Gingival redness (MGI). Bars represent comparisons with baseline. Symbols alone represent comparisons with placebo. *p < 0.0001; ‡*p* < 0.007; ▪*p* < 0.005; ≠*p* < 0.002; •*p* < 0.01; □ *p* < 0.02; +*p* < 0.03; ♦*p* < 0.05; $*p* < 0.001.

Secondary outcomes, pre- and post-treatment completion, are illustrated in Fig. 4 (and mean values documented in Table S2).

Gingival crevicular fluid volumes at both shallow and deep sites reduced with time post-therapy (Fig. 4a and b). Reductions in volume at shallow sites were small and changes only reached statistical significance at 8 months in the placebo (*p* = 0.007) and FVB (*p* < 0.02) groups, with supplementation having no effect different to that shown by the placebo group. In contrast, GCF volumes at deep sites were significantly reduced at all time points in all groups post-therapy, with reductions being consistently greater in supplementation groups (*p* < 0.002; Fig. 4b). However, the greater effect of supplementation on reducing GCF volume at deep sites was only significant at 2 months for the FVB group (*p* < 0.05).

Significant reductions in cumulative plaque scores were detected at all time points post-therapy in all groups (Fig. 4c; *p* < 0.03). No differences were evident between groups until month 8, when the reduction in plaque scores relative to baseline were significantly higher in the FV group (*p* < 0.05) compared with the placebo group.

There were progressive improvements in recession and MGI post-therapy in all three study groups (Fig. 4d and e; *p* < 0.001), but no significant differences in the magnitude of improvement between groups at any time point.

## Discussion

The current study is the first to report the impact of providing periodontitis patients with an adjunctive phytonutrient supplement during standard mechanical non-surgical periodontal therapy. We hypothesized that additional clinical benefit would result from supplementation with a juice powder concentrate (FV) and that the triple therapy (FVB) would provide enhanced outcomes over the dual supplement (FV) due to additional polyphenolic antioxidants. The rationale for such hypotheses was based upon the substantial literature base concerning the efficacy of antioxidant micronutrients in reducing extracellular oxidative stress, and their intracellular role in the down-regulation of redox-regulated pro-inflammatory gene transcription factors (reviewed by Chapple & Matthews 2007, Chapple 2009). Statistically significant additional reductions in PPD were seen at the 2-month post-therapy recall visit for FV verses placebo, providing partial fulfilment of the first hypothesis. Therapy employing the FVB supplement did not, however, provide significantly improved primary outcomes relative to the placebo group, despite the presence of additional polyphenolic micronutrients within. Hypothesis two was therefore not fulfilled and nor was hypothesis three, as the initially greater pocket depth reduction in the FV group was not sustained at 8 months post-therapy.

It was surprising not to see similar additional improvements for the FVB as the FV group, but this may be explained by the reduced bioavailability of micronutrients in this group as demonstrated by the serum β-carotene levels (Fig. 2). It may also be explained by nutrigenetic differences between groups, as polymorphisms in the BCMO1 gene (β-carotene 15,15′-monoxygenase) have been identified (Leung et al. 2009) which appear to explain, at least in part, the low conversion rates of β-carotene to its bioactive form in many individuals. The attenuation in serum β-carotene concentrations at 5 and 8 months could also have arisen due to reduced adherence to supplementation in the FVB group; however, this thesis was not supported by the supplement diary and capsule count analysis. An alternative explanation is that micronutrient absorption across the gastrointestinal tract wall may have been lower in the FVB than the FV group, or that the additional polyphenolic compounds within the FVB supplement antagonized beneficial activities of other components. Whatever the explanation, the plasma β-carotene data demonstrate reduced bioavailability of some micronutrients in the FVB group relative to FV.

Significant additional reductions in percentage sites bleeding on probing arose for the FV group at 5 months (following the increased 2-month pocket depth reductions). The cumulative plaque index data recorded in the supplementation group are also interesting, as significant additional decreases were observed at the 8-month follow-up visit for FV verses placebo, 3 months after the additional improvements in percentage sites bleeding recorded in the same group (FV). Although it could be argued that the improved plaque index in the supplement group arose due to better home care/compliance, this seems unlikely, as there were no differences between groups in plaque indices at 2- and 5-month recalls, where the most substantial reductions in plaque took place. An alternative and intriguing explanation is that additional reductions in inflammation evident at 5 months were reflected in the composition of the GCF, which in turn led to a reduced biofilm accumulation. Although this is consistent with current evidence that the inflammatory status of the gingival tissues influences plaque biofilm accumulation (Hillam & Hull 1977, Baumgartner et al. 2009) and with current consensus views on host response/biofilm inter-relationships (Tonetti & Chapple 2011), plaque re-growth studies and those analysing GCF composition would be necessary to substantiate such a thesis. A prebiotic effect upon biofilm accumulation is unlikely as capsules were swallowed and nutrients not applied locally. Any effect therefore would appear likely to be due to the influence of an altered systemic (and downstream local) host response upon the periodontal biofilm (Marsh & Devine 2011).

The healing and resolution of inflammation arising from the non-surgical mechanical therapy alone (placebo group) was substantial and consistent with the literature (van der Weijden & Timmermann 2002), with clinically significant increases in the number of closed pockets apparent at 2 months and at subsequent recalls. Moreover, there were no baseline differences between groups in circulating concentrations of vitamin E, vitamin C or β-carotene, all of which were within or close to the low end of published SI reference ranges (Young 1987). Taken together, it is perhaps surprising that any additional clinical benefit was achieved in patients who were effectively nutritionally replete and in whom non-surgical management alone was highly successful. Some of those benefits were not retained after 8 months, a finding that does not fulfil our third hypothesis and which may be explained by a number of factors, including a tendency for recalcitrant disease to develop at this stage (Quirynen et al. 2005). However, given that there is a more limited rationale to the expectation of improved therapeutic outcomes in nutritionally replete verses deplete patients (Schifferle 2009) and that non-surgical therapy was successful without adjunctive nutritional support, it is possible that more substantial clinical benefits may be realized from such dietary augmentation in nutritionally depleted periodontitis patients or in those who do not have access to dental services for periodontal therapy. The public health benefits of such an approach, in a society where obesity trends are increasing and diets are decreasing in quality (Jenzsch et al. 2009) may be substantial if proven. Indeed, there is evidence from one intervention study in patients with metabolic syndrome that inflammatory periodontal outcome measures improved following a nutritional intervention (Jenzsch et al. 2009). Such findings when allied to the impact of periodontal inflammation upon systemic inflammatory status may further impact upon chronic inflammation in the body in general (Kawashima et al. 2007, Jin et al. 2010).

There are limitations to the current study. Firstly, the study may have lacked the power to detect the beneficial effects of FV and FVB because we had no prior data upon which to base sample size estimates. Secondly, assessing protocol adherence was difficult, preventing a “per protocol” analysis. The analysis presented is an “ITT” analysis, which assumes that non-adherence will be random and thus equitable across the three groups. We did not attempt to ascertain nutrigenetic parameters which are likely to dictate “high responders” and “low responders”, largely because this science is in its infancy and the most appropriate outcome measures are unknown, with the exception of recent discoveries in relation to β-carotene converter status and polymorphisms in the BCMO1 gene (Leung et al. 2009). Thirdly, the high improvements recorded in the placebo group from mechanical debridement alone are likely to have created a ceiling effect, whereby further improvement from adjunctive therapy is less likely.

We conclude that adjunctive daily supplementation with an encapsulated FV juice powder concentrate, during non-surgical periodontal therapy, appears to offer additional initial pocket depth reductions, and subsequent additional improvements in bleeding on probing and plaque scores even in high responders to conventional therapy and in those who are not nutritionally compromised. However, outcomes seem to depend upon the serum/plasma micronutrient concentrations achieved, and this requires patient adherence and likely favourable absorption across the gastrointestinal tract wall. Further studies are necessary to assess such approaches in larger and more diverse populations, employing different supplement and therapeutic regimes. In particular, monotherapy studies (phytonutrient supplementation alone) and studies in nutritionally compromised patients are necessary to help build a clearer picture of the clinical impact of antioxidant phytonutrient supplementation upon periodontal and systemic inflammatory status.

## Supporting Information

**Table S1.** Primary clinical outcomes pre- and post-therapy (mean ± SD).

**Table S2.** Secondary clinical outcomes pre- and post-therapy (mean ± SD).

## References

[b1] Amaliya, Timmermann MF, Abbas F, Loos BG, van der Weijden GA, van Winkelhoff AJ, Winkel EG, van der Velden U (2007). Java project on periodontal diseases: the relationship between vitamin C and the severity of periodontitis. Journal of Clinical Periodontology.

[b2] Amarasena N, Ogawa H, Yoshihara A, Hanada N, Miyazaki H (2005). Serum vitamin C-periodontal relationship in community-dwelling elderly Japanese. Journal of Clinical Periodontology.

[b3] Baumgartner S, Imfeld T, Schicht O, Rath C, Persson RE, Persson GR (2009). The impact of the Stone Age diet on gingival conditions in the absence of oral hygiene. Journal of Periodontology.

[b4] Bawadi HA, Khader YS, Haroun TF, Al-Omar M, Tayyem RF (2011). The association between periodontal disease, physical activity and healthy diet among adults in Jordan. Journal of Periodontal Research.

[b5] Brock GR, Butterworth CJ, Matthews JB, Chapple IL (2004). Local and systemic total antioxidant capacity in periodontitis and health. Journal of Clinical Periodontology.

[b6] Bullon P, Morillo M, Ramirez-Tortosa MC, Quiles JL, Newman HN, Battino M (2009). Metabolic syndrome and periodontitis: is oxidative stress a common link?. Journal of Dental Research.

[b7] Ceriello A, Esposito K, Piconi L, Ihnat MA, Thorpe JE, Testa R, Boemi M, Giugliano D (2008). Oscillating glucose is more deleterious to endothelial function and oxidative stress than mean glucose in normal and type 2 diabetic patients. Diabetes.

[b8] Chapple ILC, Milward MR, Dietrich T (2007a). The prevalence of inflammatory periodontitis is negatively associated with serum antioxidant concentrations. Journal of Nutrition.

[b9] Chapple ILC (2009). Potential mechanisms underpinning the nutritional modulation of periodontal inflammation. Journal of the American Dental Association.

[b10] Chapple ILC, Brock G, Eftimiadi C, Matthews JB (2002). Glutathione in gingival crevicular fluid and its relation to local antioxidant capacity in periodontal health and disease. Journal of Clinical Pathology: Molecular Pathology.

[b11] Chapple ILC, Brock GR, Milward MR, Ling N, Matthews JB (2007b). Compromised GCF total antioxidant capacity in periodontitis: cause or effect?. Journal of Clinical Periodontology.

[b12] Chapple ILC, Landini G, Griffiths GS, Patel NC, Ward RSN (1999). Calibration of the Periotron 8000® and 6000® by polynomial regression. Journal of Periodontal Research.

[b13] Chapple ILC, Mason GM, Matthews JB, Thorpe GHG, Maxwell SRJ, Whitehead T (1997). Enhanced chemiluminescent assay for measuring the total antioxidant capacity of serum, saliva and crevicular fluid. Annals of Clinical Biochemistry.

[b14] Chapple ILC, Matthews JB (2007). The role of reactive oxygen and antioxidant species in periodontal tissue destruction. Periodontology.

[b15] Cobb CM (2002). Clinical significance of non-surgical periodontal therapy: an evidence-based perspective of scaling and root planing. Journal of Clinical Periodontology.

[b16] D'Aiuto F, Nibali L, Parkar M, Patel K, Suvan J, Donos N (2010). Oxidative stress, systemic inflammation and severe periodontitis. Journal of Dental Research.

[b17] D'Aiuto F, Parkar M, Andreou G, Suvan J, Brett PM, Ready D, Tonetti MS (2004b). Periodontitis and systemic inflammation: control of the local infection is associated with a reduction in serum inflammatory markers. Journal of Dental Research.

[b18] D'Aiuto F, Ready D, Tonetti MS (2004a). Periodontal disease and C-reactive protein-associated cardiovascular risk. Journal of Periodontal Research.

[b19] Enwonwu CO, Philips RS, Falkler WA (2002). Nutrition and oral infectious diseases: state of the science. Compendium of continuing dental education.

[b20] Esposito K, Ciotola M, Carleo D, Schisano B, Sardelli L, Di Tommaso D, Misso L, Saccomanno F, Ceriello A, Giugliano D (2008). Post-meal glucose peaks at home associate with carotid intima-media thickness in type 2 diabetes. Journal of Clinical Endocrinology and Metabolism.

[b21] Galli C, Passeri G, Macaluso GM (2011). FoxOs, Wnts and oxidative stress-induced bone loss: new players in the periodontitis arena ?. Journal of Periodontal Research.

[b22] Grant MM, Brock GR, Matthews JB, Chapple ILC (2010). Crevicular fluid glutathione levels in periodontitis and the effect of non-surgical therapy. Journal of Clinical Periodontology.

[b23] Hillam DG, Hull PS (1977). The influence of experimental gingivitis upon plaque formation. Journal of Clinical Periodontology.

[b24] Iverson C, Christiansen S, Flanagin A, Fontanarosa P, Glass R, Gregoline B, Lurie SJ, Meyer HS, Winker MA, Young RK (2007). AMA Manual of Style.

[b25] Jenzsch A, Eick S, Rassoul F, Purschwitz R, Jentsch H (2009). Nutritional intervention in patients with periodontal disease: clinical, immunological and microbiological variables during 12 months. British Journal of Nutrition.

[b26] Jin Y, Cui X, Singh UP, Chumanevich AA, Harmon B, Cavicchia P, Hofseth AB, Kotakadi V, Stroud B, Volate SR, Hurley TG, Hebert JR, Hofseth LJ (2010). Systemic inflammatory load in humans is suppressed by consumption of two formulations of dried, encapsulated juice concentrate. Molecular Nutrition Food Research.

[b27] Joshipura K, Hu FB, Manson JE, Stampfer MJ, Rimm EB, Speizer FE, Colditz G, Ascherio A, Rosner B, Spiegelman D, Willett WC (2001). The effect of fruit and vegetable intake on risk for coronary heart disease. Annals of Internal Medicine.

[b28] Joshipura KJ, Ascherio A, Manson JE, Stampfer MJ, Rimm EB, Speizer FE, Hennekens CH, Spiegleman D, Willet WC (1999). Fruit and vegetable intake in relation to risk of ischaemic stroke. Journal of the American Medical Association.

[b29] Kawashima A, Madarame T, Koike H, Komatsu Y, Wise JA (2007). Four week supplementation with mixed fruit and vegetable juice concentrates increased protective serum antioxidants and folate and decreased plasma homocysteine in Japanese subjects. Asia Pacific Journal of Clinical Nutrition.

[b30] Knekt P, Jarvinen R, Reunanen A, Maatela J (1996). Flavonoid intake and coronary mortality in Finland: a cohort study. British Medical Journal.

[b31] König J, Holtfreter B, Kocher T (2010). Periodontal health in Europe: future trends based on treatment needs and the provision of periodontal services – position paper 1. European Journal of Dental Education.

[b32] Konopka T, Król K, Kopeć W, Gerber H (2007). Total antioxidant status and 8-hydroxy-2′-deoxyguanosine levels in gingival and peripheral blood of periodontitis patients. Archivum Immunologiae et Therapiae Experimentalis (Warszawa).

[b33] Leung WC, Hessel S, Méplan C, Flint J, Oberhauser V, Tourniaire F, Hesketh JE, von Lintig J, Lietz G (2009). Two common single nucleotide polymorphisms in the gene encoding β-carotene 15,15′ –monoxygenase alter β-carotene metabolism in female volunteers. The FASEB Journal.

[b34] Linden GJ, McClean KM, Woodside JV, Patterson CC, Evand A, Young IS, Kee F (2009). Antioxidants and periodontitis in 60–70-year-old men. Journal of Clinical Periodontology.

[b35] Lobene R, Weatherford T, Ross N, Lamm R, Menaker L (1986). A modified gingival index for use in clinical trials. Clinical Preventive dentistry.

[b36] Lobene RR, Soparker PM, Newman BS (1982). Use of Dental Floss. Effect of plaque and gingivitis. Clinical Preventive Dentistry.

[b37] Marsh PD, Devine DA (2011). How is the development of dental biofilms influenced by the host. Journal of Clinical Periodontology.

[b38] Michalowicz BS, Aeppli D, Virag JG, Klump DG, Hinrichs JE, Segal NL, Bouchard TJ, Pihlstrom BL (1991). Periodontal findings in adult twins. Journal of Periodontology.

[b39] Monnier L, Mas E, Ginet E, Michel F, Villon L, Cristol JP, Colette C (2006). Activation of oxidative stress by acute glucose fluctuations compared with sustained chronic hyperglycemia in patients with type 2 diabetes. Journal of the American Medical Association.

[b40] Nishada M, Grossi SG, Dunford RG, Ho AW, Trevisan M, Genco RJ (2000). Dietary vitamin C and the risk for periodontal disease. Journal of Periodontology.

[b41] O'Keefe J, Bell D (2007). Postprandial hyperglycemia/hyperlipidemia (postprandial dysmetabolism) is a cardiovascular risk factor. American Journal of Cardiology.

[b42] O'Keefe JH, Gheewala NM, O'Keefe JO (2008). Dietary strategies for improving postprandial glucose, lipids, inflammation, and cardiovascular health. Journal of the American College of Cardiology.

[b43] Panjamurthy K, Manoharan S, Ramachandran CR (2005). Lipid peroxidation and antioxidant status in patients with periodontitis. Cellular and Molecular Biology Letters.

[b44] Plotnick GD, Corretti MC, Vogel RA, Hesslink R, Wise JA (2003). Effect of supplemental phytonutrients on impairment of the flow-mediated brachial vasoactivity after a single high fat meal. Journal of the American College of Cardiology.

[b45] Polidori MC, Stahl W, Eichler O, Niestroj I, Sies H (2001). Profiles of antioxidants in human plasma. Free Radical Biology & Medicine.

[b46] Quirynen M, Vogels R, Pauwels M, Haffajee AD, Socransky SS, Uzel NG, van Steenberghe D (2005). Initial subgingival colonization of ‘pristine’ pockets. Journal of Dental Research.

[b47] Sauvaget C, Nagano J, Hayashi M, Spencer E, Shimizu Y, Allen N (2003). Vegetables and fruit intake and cancer mortality in the Hiroshima/Nagasaki Life Span Study. British Journal of Cancer.

[b48] Schifferle RE (2009). Periodontal disease and nutrition: separating the evidence from current fads. Periodontology.

[b49] Sies H, Jones D, Fink G (2007). Oxidative stress. Encyclopedia of Stress.

[b50] Sies H, Stahl W, Sevanian A (2005). Nutritional, dietary and postprandial oxidative stress. Journal of Nutrition.

[b51] Stahl W, Sundquist AR, Hanusch M, Schwarz W, Sies H (1993). Separation of beta-carotene and lycopene geometrical isomers in biological samples. Skin Research Technology.

[b52] Sugano N, Kawamoto K, Numazaki H, Murai S, Ito K (2000). Detection of mitochondrial DNA mutations in human gingival tissues. Journal of Oral Science.

[b53] Takane M, Sugano N, Iwasaki H, Iwano Y, Shimizu N (2002). New biomarker evidence of oxidative DNA damage in whole saliva from clinically healthy and periodontally diseased individuals. Journal of Periodontology.

[b54] Tonetti MS, Chapple ILC (2011). Nutritional modulation of periodontal inflammation. In: Biological Approaches to the Development of Novel Periodontal Therapies Group 3 – Consensus of the 7th European Workshop on Periodontology. Journal of Clinical Periodontology.

[b55] Van Dyke TE (2008). The management of inflammation in periodontal disease. Journal of Periodontology.

[b56] van der Velden U, Kuzmanova D, Chapple ILC (2011). Micronutritional approaches to periodontal therapy. Journal of Clinical Periodontology.

[b57] Wei PF, Ho KY, Ho YP, Wu YM, Yang YH, Tsai CC (2004). The investigation of glutathione peroxidase, lactoferrin, myeloperoxidase and interleukin-1b in gingival crevicular fluid: implications for oxidative stress in human periodontal diseases. Journal of Periodontal Research.

[b58] van der Weijden GA, Timmermann MF (2002). A systematic review on the clinical efficacy of subgingival debridement in the treatment of chronic periodontitis. Journal of Clinical Periodontology.

[b59] Wennström JL, Tomasi C, Bertelle A, Dellasega E (2005). Full-mouth ultrasonic debridement versus quadrant scaling and root planing as an initial approach in the treatment of chronic periodontitis. Journal of Clinical Periodontology.

[b60] Young DS (1987). Implementation of SI units for clinical laboratory data. Annals of Internal Medicine.

