# Supplementary material for: Adjunctive daily supplementation with encapsulated fruit, vegetable and berry juice powder concentrates and clinical periodontal outcomes: a double-blind RCT
Source: J Clin Periodontol. 2012 Jan;39(1):62–72. doi: 10.1111/j.1600-051X.2011.01793.x (PMC3267052; doi:10.1111/j.1600-051X.2011.01793.x)
Supplement: Supplementary file 1 [file jcpe0039-0062-SD1.doc]

**Supplementary Table 1: Primary clinical outcomes pre- and post-therapy (mean + SD)**

| **Treatment** | **Pre-therapy** | **Post-therapy** | | |
| --- | --- | --- | --- | --- |
|  |  | **2-months** | **5-months** | **8-months** |
| **% Sites BOP** | | | | |
| **Placebo** | 27.7 + 11.6 | 12.7 + 11.4 | 11.8 + 11.4 | 12.4 + 13.1 |
| **FV** | 30.1+13.5 | 8+6.1 | 6.2+4.0 | 6.3+3.7 |
| **FVB** | 28 + 15.3 | 10.9 + 12.4 | 8.6 + 5.8 | 11.1 + 11.9 |
| **CAL (mm)** | | | | |
| **Placebo** | 4.85 + 0.99 | 4.6 + 0.60 | 4.6 + 0.6 | 4.7 + 0.6 |
| **FV** | 4.7 + 0.8 | 4.3 + 0.54 | 4.5 + 0.41 | 4.5 + 0.47 |
| **FVB** | 4.87 + 0.85 | 4.58 + 0.64 | 4.5 + 0.54 | 4.5 + 0.47 |
| **Pocket depth (mm)** | | | | |
| **Placebo** | 3.47 + 0.6 | 2.64 + 0.27 | 2.45 + 0.19 | 2.43 + 0.21 |
| **FV** | 3.3 + 0.4 | 2.42 + 0.23 | 2.37 +0.16 | 2.3 + 0.13 |
| **FVB** | 3.51 + 0.55 | 2.6 + 0.23 | 2.43 + 0.18 | 2.42 + 0.19 |
| All sample sizes=20 | | | | |
