# Supplementary material for: Adjunctive daily supplementation with encapsulated fruit, vegetable and berry juice powder concentrates and clinical periodontal outcomes: a double-blind RCT
Source: J Clin Periodontol. 2012 Jan;39(1):62–72. doi: 10.1111/j.1600-051X.2011.01793.x (PMC3267052; doi:10.1111/j.1600-051X.2011.01793.x)
Supplement: Supplementary file 2 [file jcpe0039-0062-SD2.doc]

**Supplementary Table 2: Secondary clinical outcomes pre- and post-therapy (mean + SD)**

| **Treatment** | **Pre-therapy** | **Post-therapy** | | |
| --- | --- | --- | --- | --- |
|  |  | **2-months** | **5-months** | **8-months** |
| **GCF volume (μl): shallow sites** | | | | |
| **Placebo** | 0.36+0.13 | 0.37+0.22 | 0.3 + 0.16 | 0.25 + 0.13 |
| **FV** | 0.28+0.19 | 0.27+0.15 | 0.26 + 0.19 | 0.24 + 0.13 |
| **FVB** | 0.3+0.15 | 0.25+0.18 | 0.21 + 0.16 | 0.19 + 0.12 |
| **GCF volume (μl): deep sites** | | | | |
| **Placebo** | 0.77 + 0.3 | 0.5 + 0.25 | 0.46 + 0.25 | 0.44 + 0.26 |
| **FV** | 0.7 + 0.3 | 0.39 + 0.22 | 0.31 + 0.16 | 0.3 + 0.2 |
| **FVB** | 0.9 + 0.3 | 0.4 + 0.19 | 0.39 + 0.2 | 0.33 + 0.15 |
| **Cumulative plaque index** | | | | |
| **Placebo** | 425 + 89 | 376 + 88 | 369 + 82 | 385 + 89 |
| **FV** | 376 + 93 | 321 + 60 | 312 + 54 | 319 + 55 |
| **FVB** | 391 + 81 | 326 + 72 | 338 + 73 | 346 + 54 |
| **MGI** | | | | |
| **Placebo** | 117+34 | 66+36 | 66 + 33 | 68 + 39 |
| **FV** | 108+26 | 56+26 | 48 + 19 | 52.5 + 20 |
| **FVB** | 105+27 | 53+24 | 44 + 22 | 48 + 23.5 |
| **Recession (mm)** | | | | |
| **Placebo** | 1.38 + 0.76 | 1.97 + 0.55 | 2.2 + 0.56 | 2.3 + 0.5 |
| **FV** | 1.4 + 0.6 | 1.87 + 0.46 | 2.1 + 0.33 | 2.2 + 0.43 |
| **FVB** | 1.37 + 0.65 | 1.98 + 0.56 | 2.0 + 0.54 | 2.1 + 0.44 |
| All sample sizes=20 | | | | |
